# The Effects of Limonin, Myo-Inositol, and L-Proline on the Cryopreservation of Debao Boar Semen

**DOI:** 10.3390/ani15152204

**Published:** 2025-07-27

**Authors:** Qianhui Feng, Yanyan Yang, Bing Zhang, Wen Shi, Yizhen Fang, Chunrong Xu, Zhuxin Deng, Wanyou Feng, Deshun Shi

**Affiliations:** 1 Guangxi Key Laboratory of Animal Breeding and Disease Control, College of Animal Science and Technology, Guangxi University, Nanning 530004, China; 2118391004@st.gxu.edu.cn (Q.F.); 2218401005@st.gxu.edu.cn (Y.Y.); 2118301041@st.gxu.edu.cn (B.Z.); 2118401004@st.gxu.edu.cn (W.S.); 2218391010@st.gxu.edu.cn (Y.F.); 2 Guangxi Work Station of Livestock & Poultry Breed Improvement, Nanning 530001, China; xp13377178849@163.com (C.X.); dengzhuxin.ccc@126.com (Z.D.); 3 School of Environmental and Life Sciences, Nanning Normal University, Nanning 530023, China

**Keywords:** limonin, myo-inositol, L-proline, freezing diluent, sperm

## Abstract

Semen cryopreservation is associated with sperm vulnerability to oxidative stress and ice crystal-induced damage, adversely affecting in vitro fertilization (IVF) success. This study aimed to investigate the effects of freezing diluent supplemented with antioxidant limonin (Lim), myo-inositol (MYO), and the ice crystal formation inhibitor L-proline (LP) through sperm motility, morphological integrity, and antioxidant capacity. The Lim (150 mM), MYO (90 mM), and LP (100 mM) significantly ameliorated the quality of post-thaw sperm in Debao boar, and combined treatment of these agents significantly enhanced sperm motility, structural integrity, and antioxidant capacity compared with individual agents (*p* < 0.05). Notably, the combined use of these agents reduced glycerol concentration in the freezing diluent from 3% to 2%. Meanwhile, the integrity of the sperm plasma membrane, acrosome membrane, and mitochondrial membrane potential was significantly improved (*p* < 0.05), and the result of IVF revealed the total cell count of the blastocysts was also greater in the 2% glycerol group (*p* < 0.05). In conclusion, the newly developed freezing diluent for semen, by adding Lim (150 mM), MYO (90 mM), and LP (100 mM), can enhance the quality of frozen–thawed Debao boar sperm and reduce the concentration of glycerol from 3% to 2% as high concentrations of glycerol can impair the quality of thawed sperm and affect in vitro fertilization outcomes. In conclusion, the improved dilution solution formulated demonstrated efficacy in enhancing the quality of porcine spermatozoa following cryopreservation and subsequent thawing.

## Simple Summary

The study enhanced the freezing diluent by incorporating antioxidants (limonin and myo-inositol) and an ice crystal inhibitor (L-proline), improving the quality and antioxidant capacity of thawed sperm. Reducing glycerol concentration to 2% maintained sperm survival rates and antioxidant enzyme levels while enhancing structural integrity and functional parameters. In vitro fertilization experiments demonstrated higher blastocyst cell counts in the low-glycerol group compared to the 3% glycerol group. These findings indicate that the improved cryopreservation medium better preserves Dabao black pig semen quality, boosting breeding efficiency and genetic resource conservation, which is beneficial for agricultural production and animal genetic material preservation.

## 1. Introduction

Debao pig is an indigenous breed native to Guangxi, China, and is renowned for its superior meat quality and distinctive genetic traits. Despite its agricultural and economic importance, the preservation of genetic traits from this breed has been hindered by the lack of effective semen cryopreservation technique, which is a critical tool in genetic conservation and artificial insemination programs.

The susceptibility of boar sperm to cryopreservation-induced stress is attributed to their elevated polyunsaturated fatty acids, which are prone to generating a significant amount of reactive oxygen species (ROS) under low-temperature conditions [1]. Elevated ROS levels can inhibit the expression of critical proteins in sperm, resulting in apoptosis and/or necrosis of sperm and causing alterations in sperm function [2,3]. Furthermore, during the freezing process, the decrease in temperature causes the liquid water molecules in the diluent to rearrange and form ice crystals, which affect the physiological activity of the sperm and result in loss of function [4,5].

Antioxidants and cryoprotectants in cryopreservation diluents are key to mitigating oxidative damage and ice crystal injury. Research has shown that adding antioxidants such as vitamin C, chlorogenic acid, Astragalus polysaccharides, and glutathione to sperm cryopreservation diluents can improve post-thaw sperm vitality and reduce oxidative damage [6,7,8]. Limonin (Lim), a compound with antioxidant, antibacterial, and antiviral activities [9], has been reported to possess an antioxidant capacity 12.5~17.2 times greater than that of vitamin C [10]. Lim can effectively inhibit the production of ROS and scavenge the free radicals [11]. Myo-inositol (MYO), abundantly present in boar seminal plasma, epididymal fluid, and seminal vesicle secretions [12,13,14], regulates sperm cell maturation, capacitation, and the acrosome reaction [15]. D-chiro-inositol has been shown to improve sperm motility in patients with asthenospermia, enhance mitochondrial membrane potential [16], and reduce ROS levels in sperm [17]. Incorporating MYO into sperm-freezing diluent has been found to enhance human sperm parameters and protect them against DNA fragmentation and apoptosis [18].

The commonly used cryoprotectant for boar semen is glycerol. However, glycerol has a high osmotic pressure, which can lead to sperm dehydration and damage the acrosome, neck, and tail of sperm, thereby affecting fertilization capacity [19]. Adel et al. found that combining guar gum and ethylene glycol or glycerol improved the antioxidant enzyme and membrane integrity of thawed sperm [20]. Additionally, partially replacing glycerol with cholesterol-loaded cyclodextrin also significantly enhanced the quality of frozen–thawed sperm [21]. These findings suggest that using other cryoprotectants in conjunction with low glycerol concentrations can effectively improve the quality of frozen–thawed sperm. L-proline (LP), a naturally occurring amino acid with osmoprotective and antioxidant properties, is commonly used as a cryoprotectant [22]. Under freezing stress, it has been shown to enhance the freeze tolerance of yeast and Drosophila [23], as well as improve the post-thaw motility of ram [24] and donkey [25] sperm.

This study investigates the effects of Lim, MYO, and LP on the cryopreservation of Debao boar semen, aiming to further optimize the cryopreservation diluent formulation.

## 2. Materials and Methods

### 2.1. Animals Used for Semen Collection

Semen was collected from the Livestock and Poultry Breeding Improvement Station in the Guangxi Zhuang Autonomous Region. In this study, semen was collected from healthy, sexually vigorous boars, approximately 2 years old (*n* = 8). Each boar was collected twice a week using the gloved-hand method, with the ejaculate collected in three fractions during each collection. Sperm with >80% motility and 85% morphologically normal sperm were used in this study (Supplementary Table S1).

### 2.2. Reagents and Extender

TCM199 (12340030, USA) and Fetal Bovine Serum (A5256701, USA) were purchased from Gibco (Waltham, MA, USA). Acridine orange staining solution (AO staining solution, CA1143, China) was purchased from Solarbio (Beijing, China), and the JC-1 staining solution (C2003S, China) was obtained from Beyotime (Shanghai, China). The total antioxidant capacity (T-AOC, A015-3-1, China), catalase (CAT, A007-1-1, China), malondialdehyde (MDA, A003-1-2, China), glutathione peroxidase (GSH-PX, A005-1-2, China), and superoxide dismutase (SOD, A001-3-2, China) assay kits were acquired from Nanjing Jiancheng Bioengineering Institute (Nanjing, China). Other chemicals and reagents were obtained from Sigma Aldrich (St. Louis, MO, USA) unless otherwise specified.

Extender I (pH = 7.2): Glucose (29.6 mg/mL), EDTA (1 mg/mL), KCl (0.6 mg/mL), sodium citrate (4.8 mg/mL), NaHCO_3_ (1 mg/mL), penicillin sodium (0.48 mg/mL), streptomycin sulfate (0.8 mg/mL), and 20% sterile egg yolk (*v*/*v*). Extender II: Glycerol is added to Extender I. The glycerol is added in different proportions according to the experiment.

### 2.3. Semen Collection and Cryopreservation

Semen was collected from a Debao boar with excellent reproductive performance, stored in a 37 °C incubator, and transported to the cryopreservation facility within 30 min for subsequent cryopreservation analysis. Normal semen appeared milky white or pale yellow and had a slight fishy odor. Subsequently, the semen was first equilibrated for 30 min at 17 °C in a constant-temperature box and then centrifuged at 2500 rpm for 15 min (JIDI-20R, JIDI Instrument, Guangzhou, China) to discard the supernatant. The post-centrifugation sperm was promptly resuspended in Cryopreservation Extender I to a density of 2 × 10^9^ sperm/mL within 10 min. It was then equilibrated at 5 °C for 3.5 h by adding an equal volume of Cryopreservation Extender II to achieve a final concentration of 1 × 10^9^ sperm/mL and subsequently placed in a 4 °C incubator for 30 min. The well-mixed sperm extender was filled into 0.25 mL straws, which were transferred into the chamber of a programmable controlled-rate freezer (Digitcool Alpha, IMV, L’Aigle, France). The cooling protocol was programmed as follows: −2 °C/min from 4 °C to 1 °C, −30 °C/min from 1 °C to −25 °C, and −30 °C/min from −25 °C to −140 °C. Finally, the straws were plunged into liquid nitrogen and stored for 30 days [26].

### 2.4. Assessment of Frozen–Thawed Semen Motility, Morphology, and Kinematics

Sperm quality analysis was performed using a CEROS II (IVM Company, L’Aigle, France) Computer-Assisted Sperm Analyzer (CASA) to evaluate sperm vitality, morphology, and kinematic parameters. Thawed semen was placed on a pre-warmed glass slide. A 40× objective was used to examine 10 fields per sample, with approximately 200 sperm observed under magnification. The CASA system settings were as follows: 45 frames required, frame rate of 60 Hz, minimum cell contrast of 46, minimum cell size of 7 pixels, chamber depth of 20 μm, straightness (STR) threshold of 80%, average path velocity (VAP) threshold of 50 μm/s, low VAP cutoff of 20 μm/s, and low straight-line velocity (VSL) cutoff of 30 μm/s. Other parameters also include curvilinear velocity (VCL, µm/s), linearity (LIN,%), wobble (WOB,%), beat cross frequency (BCF, Hz), and straight-line distance (DSL,μm/s) [27].

### 2.5. Plasma Membrane Integrity Assessment

Take 100 μL of the thawed semen and place it into 1 mL of hypotonic solution (incubated at 37 °C for 5 min). After thoroughly mixing, incubate it in a water bath at 37 °C for 30 min. Drop 15 μL of the incubated semen onto a cell-counting plate and observe the curvature of the sperm tails under a 400× optical microscope. Sperm with intact plasma membranes will swell due to water absorption in the hypotonic solution, and their tails will be curved. For each sample, 200 sperm were counted, and the experiment was repeated five times. The percentage of sperm with bent tails was calculated as the proportion of bent-tailed sperm to the total number of sperm counted (Supplementary Figure S1) [28].

### 2.6. Acrosome Integrity Assessment

Sperm were stained and observed using PNA-FITC fluorescent dye with a phase-contrast microscope. Each group was repeated thrice, with more than 200 sperm counted each time. The percentage of sperm exhibiting no green fluorescence, indicative of intact acrosomal membranes, was statistically determined among the total sperm counted (Supplementary Figure S2) [29].

### 2.7. Nuclear DNA Integrity Assessment

Sperm were stained with 0.01% AO staining solution for observation under a fluorescence microscope (400×). Sperm with intact nuclear DNA were stained with uniform yellow–green fluorescence. In contrast, sperm with incomplete nuclear DNA had condensed chromatin, and the nucleus fragmented into tiny dots and was stained with green granules of varying sizes and dense staining. Each group was repeated three times, with more than 200 sperm counted each time; the percentage of sperm with green fluorescence was statistically calculated as the proportion of the total number of counted sperm (Supplementary Figure S3) [30].

### 2.8. Measurement of Sperm Mitochondrial Activity

Sperm samples were stained with JC-1 dye and immediately observed under a fluorescence microscope. Normal sperm, characterized by high mitochondrial membrane potential, exhibited red fluorescence, whereas damaged sperm, with low mitochondrial membrane potential, displayed green fluorescence. Each experiment was repeated three times, with more than 200 sperm counted in each repetition (Supplementary Figure S4) [31].

### 2.9. Detection of Antioxidant Capacity of Post-Thaw Sperm

In the present study, the concentrations of T-AOC, indicative of antioxidant capacity GSH-PX, SOD, CAT, and oxidative stress marker MDA in semen samples, were measured using an enzyme-labeling instrument. The specific detection methods were as follows: T-AOC was detected at a wavelength of 593 nm, with results expressed in mM/L; GSH-PX was detected at a wavelength of 405 nm, with results expressed in μmol/L; SOD was detected at a wavelength of 450 nm, with results expressed in U/mL; CAT was detected at a wavelength of 405 nm, with results expressed in U/mL; and MDA was detected at a wavelength of 532 nm, with results expressed in U/mL. For each assay, standard curves were established prior to measurement to ensure the accuracy of the results [28].

### 2.10. Sperm Ultrastructure

Scanning Electron Microscopy (SEM) Sample Preparation: Thawed sperm was washed and fixed with 2.5% glutaraldehyde in 0.1 M PBS (pH 7.4) at room temperature for 2 h. After postfixation with 1% osmium tetroxide, the samples were dehydrated in a graded ethanol series (30% to 100%) and treated with isoamyl acetate. Finally, the samples were dried, coated with gold, and analyzed using a Hitachi SU8100 SEM (Chiyoda, Japan). Images were acquired at an acceleration voltage of 5–10 kV, with magnifications ranging from 10,000×, focusing on the head region, acrosome, and membrane integrity. Approximately 200 sperm per group were analyzed [32].

Transmission Electron Microscopy (TEM) Sample Preparation: Thawed sperm was washed with 0.1 M PBS (pH 7.4) and fixed with 2.5% glutaraldehyde in the same buffer at room temperature for 2 h. After post-fixation with 1% osmium tetroxide, the samples were dehydrated in a graded ethanol series and embedded in 812 embedding resin. Ultrathin sections (60–80 nm) were cut and mounted on copper grids. Sections were contrast-stained with uranyl acetate and lead citrate. A Hitachi HT-7700 TEM (Tokyo, Japan) was used to acquire images at 100,000× magnification, examining the ultrastructural integrity of sperm cells, including the head region, nucleus, acrosomal area, mitochondria, and plasma membrane. Approximately 200 sperm per group were analyzed [33].

### 2.11. In Vitro Fertilization

Pig ovaries were collected from a local slaughterhouse and transported to the laboratory in sterile saline solution at approximately 30 °C. Cumulus–oocyte complexes were selected and matured in vitro for 44 h. Mature oocytes were treated with hyaluronidase to remove cumulus cells. Fifteen oocytes were placed in 50 μL fertilization solution droplets. Thawed sperm were added to the fertilization medium and incubated for 1 h in an incubator at 38.5 °C. The mixture was centrifuged at 1500 rpm for 5 min, and the sperm concentration was adjusted to 1 × 10^6^ sperm/mL. Sperm and oocytes were co-cultured in a 5% CO_2_ incubator at 38.5 °C with high humidity for 6 h. Subsequently, the zygotes were cultured in 30 μL PZM-3 medium droplets [34].

### 2.12. Experimental Design

The study included five experimental groups to investigate the effects of different additives and glycerol concentrations on post-thaw sperm quality. The Lim group (0 mM, 50 mM, 100 mM, 150 mM, and 200 mM), the MYO group (0 mM, 30 mM, 60 mM, 90 mM, and 120 mM), the LP group (0 mM, 50 mM, 100 mM, and 150 mM), the combined addition group (Lim: 150 mM, MYO: 90 mM, and LP: 100 mM), and the group with different glycerol concentrations (0%, 1%, 2%, and 3%) were all supplemented with 150 mM Lim, 90 mM MYO, and 100 mM LP. Moreover, except for the different glycerol concentration group, all other groups contained 3% glycerol in the cryoprotectant solution. Sperm ultrastructure and IVF experiments were conducted using glycerol groups (0%, 2%, and 3%). Each group consisted of three replicates.

### 2.13. Statistical Analysis

To determine whether the data conformed to a normal distribution, the Shapiro–Wilk test was applied. The data were analyzed using the general linear model procedure (ANOVA), and Tukey’s Honestly Significant Difference (HSD) test was performed for multiple comparisons between groups using SPSS 27.0 software. The results are expressed as the mean ± standard error (Mean ± SEM). Graphs were generated using GraphPad Prism 9.5. Different letters indicate statistically significant differences (*p* < 0.05).

## 3. Results

### 3.1. Effects of Lim Supplementation on the Freezing Diluent on Boar Sperm Cryosurvival

**Figure 1:**
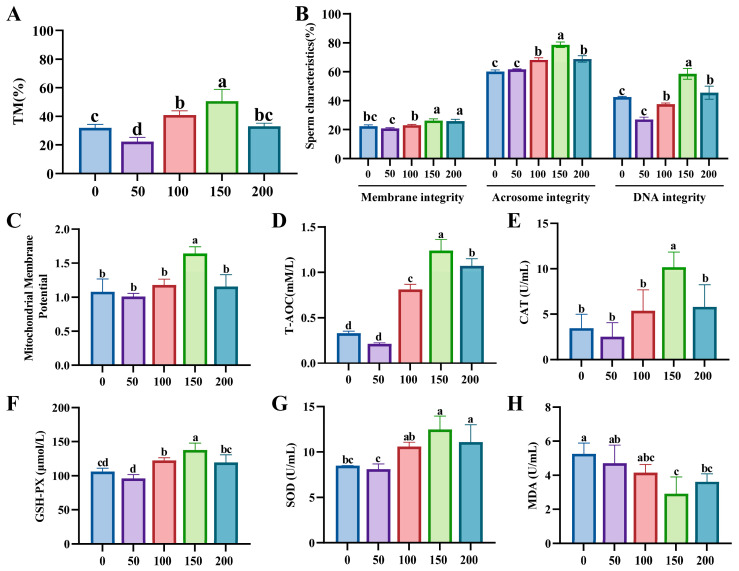
Effects of limonin (Lim; 0, 50, 100, 150, and 200 mM) supplementation on the freezing diluent on boar sperm cryo-survival. (**A**) Total motility (TM) of frozen–thawed sperm. (**B**) Morphology and structure of frozen–thawed sperm. (**C**) Mitochondrial membrane potential level of sperm of frozen–thawed sperm. (**D**) Total antioxidant capacity (T-AOC) level of frozen–thawed sperm. (**E**) Catalase (CAT) level of frozen–thawed sperm. (**F**) Glutathione peroxidase (GSH-PX) level of frozen–thawed sperm. (**G**) Superoxide dismutase (SOD) level of frozen–thawed sperm. (**H**) Malondialdehyde (MDA) level of frozen–thawed sperm. Different lowercase letters indicate statistical differences at *p*  <  0.05.

Among all Lim addition groups, only the 100 mM and 150 mM groups showed significantly higher total motility percentages compared to the control group (0 mM), while the 50 mM group exhibited significantly lower total motility than the control (*p* < 0.05, Figure 1A). Additionally, the plasma membrane integrity in the 150 mM group was significantly higher than the other groups except for the 200 mM group (*p* < 0.05). Both the acrosomal membrane and nuclear DNA integrity in the 150 mM group were also significantly higher than other groups (*p* < 0.05, Figure 1B). Meanwhile, compared to the control group, the 150 mM group exhibited a significant decrease in the percentage of curved tails, distal and proximal plasmic drop (*p* < 0.05), as well as significant improvements in DSL, WOB, LIN, and VSL (*p* < 0.05, Supplementary Table S2).

Furthermore, the mitochondrial membrane potential, T-AOC, CAT, and GSH-PX levels of the 150 mM group significantly increased compared to other groups (*p* < 0.05, Figure 1C–F). The SOD level of 150 mM and 200 mM groups increased compared to controls (*p* < 0.05), while the 50 and 100 mM groups were not different from the control group (*p* > 0.05, Figure 1G). Additionally, the MDA level of 150 mM and 200 mM groups significantly declined compared to control group (*p* < 0.05, Figure 1H), while there was no significant difference in the other groups (*p* > 0.05, Figure 1H). In conclusion, the addition of 150 mM Lim significantly enhanced the quality of sperm after freezing.

### 3.2. Effects of MYO Supplementation on the Freezing Diluent on Boar Sperm Cryosurvival

**Figure 2:**
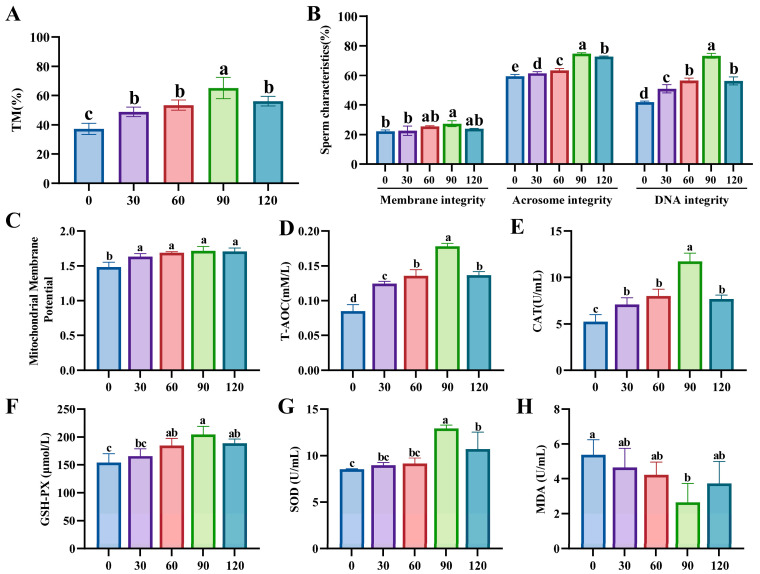
Effects of myo-inositol (MYO; 0, 30, 60, 90, and 120 mM) supplementation on the freezing diluent on boar sperm cryo-survival. (**A**) Total motility (TM) of frozen–thawed sperm. (**B**) Morphology and structure of frozen–thawed sperm. (**C**) Mitochondrial membrane potential level of sperm of frozen–thawed sperm. (**D**) Total antioxidant capacity (T-AOC) level of frozen–thawed sperm. (**E**) Catalase (CAT) level of frozen–thawed sperm. (**F**) Glutathione peroxidase (GSH-PX) level of frozen–thawed sperm. (**G**) Superoxide dismutase (SOD) level of frozen–thawed sperm. (**H**) Malondialdehyde (MDA) level of frozen–thawed sperm. Different lowercase letters indicate statistical differences at *p * <  0.05.

The addition of MYO to the freezing diluent significantly increased the percentage of total motility after thawing, with the 90 mM group showing markedly higher percentage compared to other addition groups (*p* < 0.05, Figure 2A). Figure 2B shows that the 90 mM group significantly enhances sperm plasma membrane integrity compared to the control group (*p* < 0.05) and also significantly improves sperm acrosome and nuclear DNA integrity compared to other groups (*p* < 0.05). Additionally, only the 90 mM and 120 mM MYO addition groups exhibited significant differences in DSL parameters compared to the control group (*p* < 0.05, Supplementary Table S3), but no significant differences were observed in other kinematic parameters and morphology parameters across all groups (*p* > 0.05, Supplementary Table S3).

Moreover, after MYO addition, the level of mitochondrial membrane potential significantly increased (*p* < 0.05, Figure 2C). Compared to the control group, the level of T-AOC and antioxidant enzymes including CAT, GSH-PX, and SOD were significantly increased in the 90 mM group (*p* < 0.05, Figure 2D–G). Notably, the MDA level of 90 mM groups declined compared to controls (*p* < 0.05, Figure 2H). In general, the 90 mM addition group exhibited the most significant cryosurvival effect.

### 3.3. Effects of LP Supplementation on the Freezing Diluent on Boar Sperm Cryosurvival

**Figure 3:**
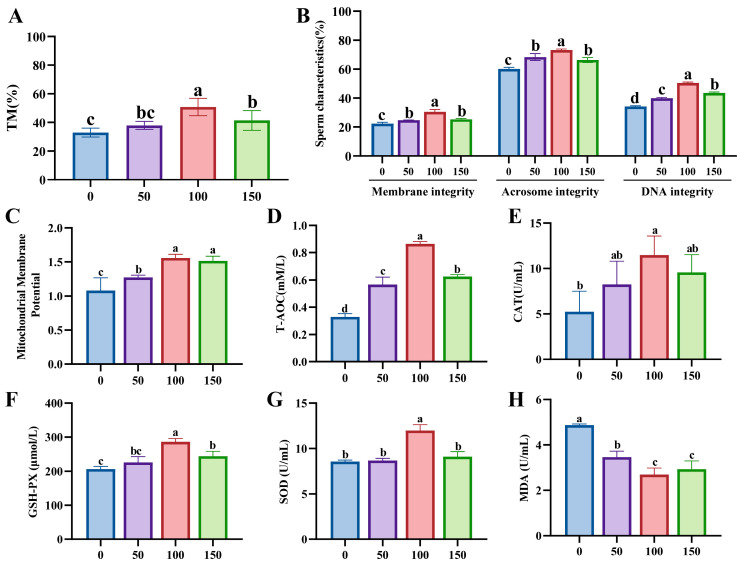
Effects of L-proline (LP; 0, 50, 100, and 150 mM) supplementation on the freezing diluent on boar sperm cryo-survival. (**A**) Total motility (TM) of frozen–thawed sperm. (**B**) Morphology and structure of frozen–thawed sperm. (**C**) Mitochondrial membrane potential level of sperm of frozen–thawed sperm. (**D**) Total antioxidant capacity (T-AOC) level of frozen–thawed sperm. (**E**) Catalase (CAT) level of frozen–thawed sperm. (**F**) Glutathione peroxidase (GSH-PX) level of frozen–thawed sperm. (**G**) Superoxide dismutase (SOD) level of frozen–thawed sperm. (**H**) Malondialdehyde (MDA) level of frozen–thawed sperm. Different lowercase letters indicate statistical differences at *p*  <  0.05.

The percentage of total motility of the 100 mM group was significantly higher than that of the control group after LP addition (*p* < 0.05, Figure 3A). As shown in Figure 3B, compared to the control group, the integrity of the plasma membrane, acrosome membrane, and nuclear DNA in thawed sperm was significantly increased in the group of 100 mM LP (*p* < 0.05). Furthermore, compared to control group, the kinematic parameter of VAP was significantly increased in the 100 mM group, and the percentage of the distal plasmic drop was significantly decreased in the 100 mM group (*p* < 0.05, Supplementary Table S4).

LP significantly enhances the level of mitochondrial membrane potential, and the 100 mM and 200 mM groups showed higher percentage than the other addition groups (*p* < 0.05, Figure 3C). Compared to the control group, the levels of T-AOC, CAT, GSH-PX, and SOD were significantly elevated in the 100 mM group (*p* < 0.05, Figure 3D–G), and the levels of MDA in the MYO addition groups were significantly lower than the control group (*p* < 0.05, Figure 3H). In conclusion, the addition of 100 mM Lim significantly enhanced the quality of sperm after freezing.

### 3.4. Effects of Combined Addition on the Freezing Diluent on Boar Sperm Cryosurvival

**Figure 4:**
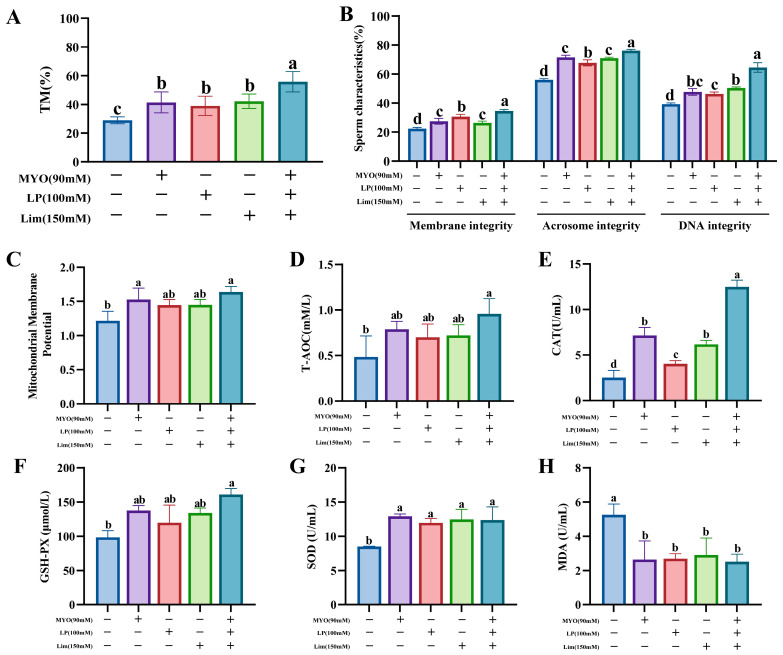
Effects of combined addition (Lim: 150 mM, MYO: 90 mM, and LP: 100 mM) on the freezing diluent on boar sperm cryo-survival. (**A**) Total motility (TM) of frozen–thawed sperm. (**B**) Morphology and structure of frozen–thawed sperm. (**C**) Mitochondrial membrane potential level of sperm of frozen–thawed sperm. (**D**) Total antioxidant capacity (T-AOC) level of frozen–thawed sperm. (**E**) Catalase (CAT) level of frozen–thawed sperm. (**F**) Glutathione peroxidase (GSH-PX) level of frozen–thawed sperm. (**G**) Superoxide dismutase (SOD) level of frozen–thawed sperm. (**H**) Malondialdehyde (MDA) level of frozen–thawed sperm. Different lowercase letters indicate statistical differences at *p*  <  0.05.

The combined addition of Lim, MYO, and LP significantly improved the percentage of total motility compared to the control group and individual addition groups (*p* < 0.05, Figure 4A). As shown in Figure 4B, the combined addition group had significantly higher membrane, acrosome, and nuclear DNA integrity rates than the control and individual addition groups (*p* < 0.05). The percentage of proximal plasmic drop was lower in the combined addition group than in the control and Lim addition groups (*p* < 0.05, Supplementary Table S5). In kinematic parameters, the combined addition group showed significantly improvement in DSL, WOB, and LIN compared to the control group (*p* < 0.05), but other kinematic parameters and morphology parameters showed no significant differences (*p* > 0.05, Supplementary Table S5).

Compared with the control group, furthermore, the combined addition group exhibited significant enhancements in mitochondrial membrane potential and T-AOC (*p* < 0.05, Figure 4C,D); simultaneously, the levels of CAT, GSH-Px, and SOD were markedly elevated (*p* < 0.05, Figure 4E–G), while MDA was significantly reduced (*p* < 0.05, Figure 4H). Notably, combined addition of Lim, MYO, and LP exhibited partial additive effects, such as TM, sperm characteristics, and CAT (Figure 4A,B,E). When comparing among individual addition groups, however, no significant differences were observed in mitochondrial membrane potential, T-AOC, GSH-Px, SOD, and MDA (*p* > 0.05, Figure 4C,D,F,H). Therefore, comprehensive analysis indicates that the combined addition of Lim, MYO, and LP is more effective for sperm cryopreservation than individual addition.

### 3.5. Effects of Different Concentrations of Glycerol on the Freezing Diluent on Boar Sperm Cryosurvival

**Figure 5:**
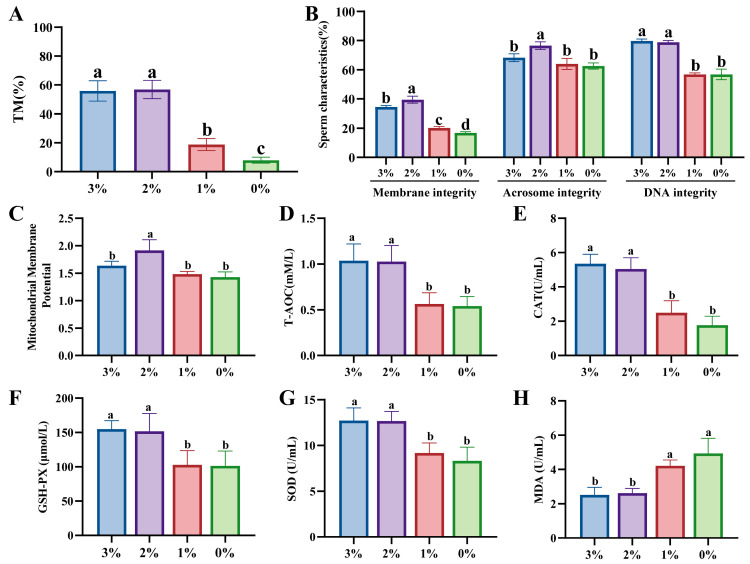
Effects of different concentrations of glycerol (0%, 1%, 2%, and 3%) on the freezing diluent on boar sperm cryo-survival. (**A**) Total motility (TM) of frozen–thawed sperm. (**B**) Morphology and structure of frozen–thawed sperm. (**C**) Mitochondrial membrane potential level of sperm of frozen–thawed sperm. (**D**) Total antioxidant capacity (T-AOC) level of frozen–thawed sperm. (**E**) Catalase (CAT) level of frozen–thawed sperm. (**F**) Glutathione peroxidase (GSH-PX) level of frozen–thawed sperm. (**G**) Superoxide dismutase (SOD) level of frozen–thawed sperm. (**H**) Malondialdehyde (MDA) level of frozen–thawed sperm. Different lowercase letters indicate statistical differences at *p*  <  0.05.

The following experiments were conducted using cryopreserved semen supplemented with glycerol (0%, 1%, 2%, and 3%) and containing 150 mM Lim, 90 mM MYO, and 100 mM LP. Figure 5A indicates that there is no significant difference in the percentage of total motility between the 2% group and 3% group (*p* > 0.05), while there is significantly higher total motility than in the 0% and 1% groups (*p* < 0.05, Figure 5A). Additionally, the plasma and acrosome membrane integrity in the 2% group was significantly superior to those in the other groups (*p* < 0.05, Figure 5B). There was no significant difference in nuclear DNA integrity between the 2% and 3% groups (*p* > 0.05), while both groups exhibited significantly higher integrity than the 0% and 1% groups (*p* < 0.05, Figure 5B). Regarding morphology and kinematic parameters, the 2% group showed no significant differences compared to the 3% group (*p* > 0.05, Supplementary Table S6).

As shown in Figure 5C, the mitochondrial membrane potential in the 2% group was significantly higher than that in the other groups (*p* < 0.05). Furthermore, there were no significant differences in the levels of T-AOC, CAT, GSH-PX, SOD, and MDA between the 2% and 3% groups (*p* > 0.05, Figure 5D–H). However, both groups had significantly higher levels than the 0% and 1% groups (*p* < 0.05, Figure 5D–H). Thus, the addition of 0.15 mol/L Lim, 0.09 mol/L MYO, and 100 mol/L LP to the semen-freezing diluent can reduce glycerol concentration from 3% to 2%.

### 3.6. Effects of Different Concentrations of Glycerol on Post-Thaw Boar Sperm Ultrastructure and Fertilization Ability

**Figure 6:**
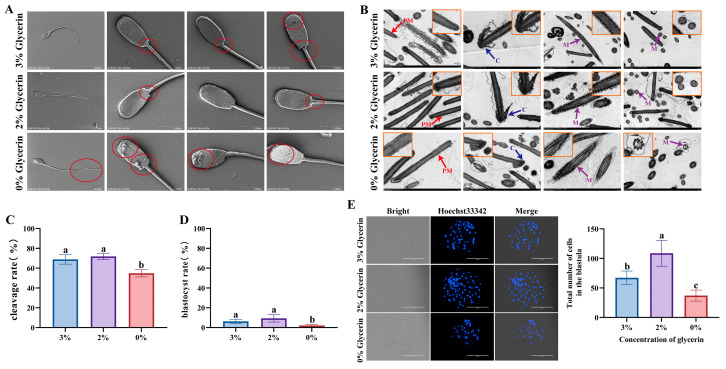
Effects of different concentrations of glycerol (0%, 2%, and 3%) on the freezing diluent on post-thaw boar sperm ultrastructure and fertilization ability. (**A**) SEM image of post-thawed boar sperm. Red circled areas in the images correspond to sites of structural defects in sperm. (**B**) TEM image of post-thawed boar sperm. PM, plasma membrane; C, acrosomal cap; M, mitochondrial sheath. Orange-boxed regions in the images indicate localized enlargements for detailed observation. (**C**) Cleavage rate after IVF with post-thawed boar sperm. (**D**) Blastocyst rate after IVF with post-thawed boar sperm. (**E**) Total number of cells per blastocyst after IVF with post-thawed boar sperm, scale bar: 200 μm. Different lowercase letters indicate statistical differences at *p*  <  0.05.

As shown in Figure 6A, the 2% group exhibited structurally intact sperm: the head displayed a smooth ellipsoidal shape with a clear and tight connection between the acrosomal membrane and plasma membrane, presenting even edges; no surface contaminants were observed on the plasma membrane; the neck junction showed minor damage but no significant swelling; and the tail remained intact. In contrast, the 3% group retained a distinct boundary between the acrosomal and plasma membranes, with the acrosomal structure tightly connected to the head plasma membrane. However, compared to the 2% group, the head plasma membrane showed slight damage with minor surface contaminants, the neck junction exhibited more severe damage, and the tail displayed noticeable bending. The 0% group, however, demonstrated marked abnormalities: the sperm head collapsed, the acrosomal membrane was damaged with vesicular outer membrane formation, and the boundary with the plasma membrane became indistinct. The plasma membrane exhibited overall wrinkling, roughness, and vesicular structures with significantly increased surface contaminants. Additionally, the neck junction showed swelling and damage, and the tail presented obvious fractures.

Figure 6B illustrates that the 2% group had intact plasma membranes and acrosomal caps, with clear cross-sectional views of the 9 + 2 microtubule structure and uniform internal microtubule distribution. The mitochondrial sheath in the midpiece of the tail maintained a regular circular shape with even distribution. Compared to the 2% group, the 3% group showed minor differences: slight damage to the acrosomal and plasma membranes, although the 9 + 2 structure cross-section remained clear with uniform microtubule distribution. However, the mitochondrial sheath in the tail exhibited heterogeneity in morphology and size. The 0% group, in stark contrast, displayed severe abnormalities, with obvious damage to the plasma membrane and acrosomal cap. Both cross-sectional and longitudinal views of the 9 + 2 structure appeared blurred, and the microtubule distribution was disordered.

Following in vitro fertilization with post-thaw sperm, cleavage rate at 2 days (Figure 6C) and blastocyst rate at 7 days (Figure 6D) were significantly elevated in the 2% and 3% glycerol groups relative to the 0% group (*p* < 0.05). Further analysis showed that the 2% group exhibited significantly higher total number of cells per blastocyst than the 3% group (*p* < 0.05, Figure 6E). In summary, the 2% glycerol group exhibited superior sperm integrity and IVF performance.

## 4. Discussion

Although the first successful sperm cryopreservation was over 60 years ago, current methods yield about 50% post-thaw viability for most species (pig, 30–40%) [19,35]; more importantly, frozen semen yields farrowing rates of only 75–80% by artificial insemination, markedly lower than those achieved with fresh semen (over 90%) [36]. To ensure minimal success, the use of cryoprotectants and antioxidants remains the conventional strategy against cryoinjury.

Lim exhibits antioxidant activity in A549 cells by reducing ROS, thereby enhancing viability [37]. Additionally, Lim suppresses ROS generation and apoptosis in embryos, thereby enhancing embryo quality [38]; however, no studies on sperm (particularly frozen sperm) have been reported. MYO enhances sperm motility and preserves structural integrity by attenuating oxidative stress [39,40]. In patients with oligo-astheno-teratozoospermia, MYO enhances sperm motility, and mitochondrial membrane potential, and reduces apoptosis [41]. Concurrently, it elevates antioxidant enzymes (GSH and SOD), decreases oxidative markers (MDA and ROS), and critically attenuates DNA damage of post-thawed sperm [42,43]. LP acts as a multifunctional cryoprotectant by preventing ice formation, stabilizing osmotic balance, scavenging ROS, and preserving membrane–protein integrity [40]. Abdelnour and colleagues demonstrated that supplementation with 2 mM LP significantly enhances the quality of cryopreserved–thawed goat spermatozoa by regulating sperm redox homeostasis [22]. Our study demonstrated that Lim, MYO, and LP exhibit antioxidant and cryoprotective effects in sperm preservation, as evidenced by reduced post-thaw MDA levels and elevated GSH-Px, SOD, and T-AOC activities when individually added to freezing extenders, alongside improved structural integrity and enhanced DNA stability.

The optimal MYO concentration in our Debao boar freezing extender (90 mM) seems higher than previously reported: 0.5 mg/mL (~2.78 mM) in Duroc boars [42], 5 μM in commercial goats [44], 3 mg/mL (~16.65 mM) in Holstein bulls [45], and 22 mM in humans [46]. Notably, this discrepancy likely arises from species differences and, more likely, variations in the base extender composition and the final sperm concentration used. The referenced studies employed final sperm concentrations of 1 × 10^8^ (Duroc boars) [42], 1.25 × 10^8^ (commercial goats) [44], 6.25 × 10^6^ (Holstein bulls) [45] and 0.75 × 10^6^ sperm/mL (humans) [46], contrasting with our 1 × 10^9^ sperm/mL. To enhance membrane stability and support cellular antioxidant capacity, the optimal MYO concentration in semen-freezing extenders should be adjusted for species differences and the final sperm concentration used. Similarly, the optimal concentration of L-proline to be added to sperm freezing extenders differs markedly owing to the three factors mentioned above, for example, 10 mM [47] or 75 mM [48] in Duroc boars, 2 mM in Laoshan goat [49], 60 mM in buffalo bull [50], and 40 mM in donkey [51]; it should be emphasized, concurrently, that interspecies variations in spermatozoal response to oxidative stress warrant particular attention.

Furthermore, the combined addition of Lim, MYO, and LP significantly increased sperm TM, characteristic parameters, and CAT levels compared to individual groups, likely due to synergistic interactions among the three agents. During semen cryopreservation, sperm were exposed not only to oxidative stress but also to cryo-injury [52]. The combined use of antioxidants and cryoprotectants significantly enhances sperm cryotolerance, reducing cryoinjuries. The underlying mechanisms may involve the following: antioxidants can protect membranes and maintain DNA integrity by inhibiting lipid peroxidation and reducing MDA levels. Meanwhile, cryoprotectants can reduce the formation of ice crystals and slow down the changes in osmotic pressure, thereby reducing the production of ROS and alleviating sperm damage caused by oxidative stress [42,49].

Glycerol, a permeable cryoprotectant, plays an indispensable role in sperm cryopreservation. However, its significantly slower permeation through the cell membrane compared to water can induce osmotic toxicity, resulting in poor frozen sperm quality [53]. Furthermore, high glycerol concentrations can impact sperm osmotic pressure and alter the structure and fluidity of the sperm membrane [54,55,56]. Notably, boar sperm, characterized by low cholesterol content and high polyunsaturated fatty acid levels, exhibit poor tolerance to glycerol [57]. Additionally, glycerol cannot ameliorate the damage to mitochondria during the freezing and thawing processes [58]. It affects in vitro fertilization outcomes in poultry [56,59], sheep [47,49], pigs [58], and horses [60], thereby hindering the production and promotion of cryopreserved sperm. Currently, numerous studies have demonstrated the addition of non-permeable cryoprotectants—including sugars, amino acids, proteins, lipoproteins, and other macromolecules—to porcine sperm cryodiluents, aiming to partially or completely replace glycerol [61,62,63,64,65]. Furthermore, LP can lower the freezing point and reduce ice crystal formation. Compared to glycerol, LP exhibits superior membrane stabilization effects [66], enhancing sperm resistance to dehydration and deformation during slow freezing, improving motility, and ultimately leading to greater recovery potential [67]. This also provides the feasibility of combining Lim, MYO, and LP to further reduce glycerol concentrations.

Our results found that the glycerol concentration in semen cryopreservation diluent can be reduced from 3% to 2% by adding Lim, MYO, and LP simultaneously. Microscopic observations of sperm showed that the integrity of the plasma membrane and acrosomal membrane was higher in the 2% group, and the mitochondrial sheath structure was intact. Damage to the plasma membrane during the semen cryopreservation process may lead to the leakage of intracellular components such as metabolic enzymes, acrosomal enzymes, and ATP, resulting in decreased motility and quality of thawed semen [68,69]. Additionally, sperm with structural defects, such as DNA damage, acrosomal membrane damage, and tail fragmentation, are detrimental to early embryonic development following in vitro fertilization [70]. Furthermore, reducing the glycerol concentration in boar semen cryopreservation diluent significantly enhances the in vitro fertilization capacity of sperm [21]. The cell number of blastocysts is a critical indicator for assessing embryo quality. Studies have demonstrated that blastocysts with a higher cell number exhibit enhanced developmental potential and increased implantation success rates [71]. The results of this study indicate that there was no significant difference in cleavage rate and blastocyst rate between the 2% glycerol group and the 3% group; the total number of per blastocyst cells, however, was higher in the 2% group compared to both the 3% and 0% glycerol concentration groups, suggesting more significant embryonic developmental potential.

## 5. Conclusions

Lim, MYO, and LP exhibit cryoprotective effects on boar semen. Adding 150 mM Lim, 90 mM MYO, and 100 mM LP to the semen-freezing extender significantly improves the freeze–thaw quality of Debao pig sperm by enhancing sperm motility, structural integrity, and antioxidant capacity. Additionally, the combined use of these additives reduces the glycerol concentration in the freezing extender while improving the quality of blastocysts formed after fertilization.

## Data Availability

No data were deposited in an official repository. All data generated during this study are available from the corresponding author upon request.

## Supplementary Materials

The following supporting information can be downloaded at https://www.mdpi.com/article/10.3390/ani15152204/s1. Table S1: Morphology and kinetic parameters of pre-freeze sperm; Table S2: Effects of different concentrations of Lim on morphology and kinematic parameters of frozen–thawed sperm; Table S3: Effects of different concentrations of MYO on morphology and kinematic parameters of frozen–thawed sperm; Table S4: Effects of different concentrations of LP on morphology and kinematic parameters of frozen–thawed sperm; Table S5: Effects of three-drug combination addition on morphology and kinematic parameters of frozen–thawed sperm; Table S6: Effects of different concentrations of glycerol in combination addition on morphology and kinematic parameters of frozen–thawed sperm; Figure S1: Plasma membrane integrity assessment; Figure S2: Acrosome integrity assessment; Figure S3: Nuclear DNA integrity assessment; Figure S4: FT Sperm mitochondrial membrane potential.
