# Supplementary material for: The Effects of Limonin, Myo-Inositol, and L-Proline on the Cryopreservation of Debao Boar Semen
Source: Animals (Basel). 2025 Jul 27;15(15):2204. doi: 10.3390/ani15152204 (PMC12345456; doi:10.3390/ani15152204)
Supplement: Supplementary file 1 [file animals-15-02204-s001.zip › Supplementary Materials-1.pdf]

Supplementary Materials

Table S1. Morphology and kinetic parameters of pre-freeze sperm (Mean ± SEM, n=8)

	pre-freeze sperm
TM(%)	86.9±2.00
Curved tails(%)	5.7 ±0.46
Coiled tails(%)	1.5±0.32
Distal plasmic drop(%)	1.83±0.22
Proximal plasmic drop(%)	0
VCL(μ m/s)	313.28±3.42
BCF（Hz）	31.41 ±0.80
DSL(μ m/s)	13.62 ±0.44
WOB(%)	42.26±1.04
VAP(μ m/s)	109.55 ±2.44
LIN(%)	18.93±0.94
VSL(μ m/s)	77.16±7.82

Table S2. Effects of different concentrations of Lim on morphology and kinetic parameters of frozen-thawed sperm (Mean ± SEM, n=8)

	0(mM)	50(mM)	100(mM)	150(mM)	200(mM)
Curved tails(%)	7.50 ±1.92^{ab}	9.30 ±2.17^{a}	5.40 ±2.57^{bc}	3.80 ±0.85^{c}	9.63 ±1.00^{a}
Coiled tails(%)	0.47 ±0.50^{b}	0.63 ±0.31^{b}	1.70 ±0.40^{a}	0.63 ±0.29^{b}	0.73 ±0.21^{b}
Distal plasmic drop(%)	7.90±2.25^{a}	9.67±0.68^{a}	4.67±0.81^{b}	4.40±1.30^{b}	8.60±1.21^{a}
Proximal plasmic drop(%)	12.00 ±3.50^{b}	15.03 ±4.23^{b}	1.57 ±0.12^{c}	1.17 ±1.01^{c}	23.07 ±6.79 ^{a}
VCL(μ m/s)	109.52±8.34^{b}	138.26 ±4.26^{a}	114.80±10.52^{b}	122.20 ±8.89^{ab}	118.08 ±7.84^{b}
BCF（Hz）	42.67 ±1.54^{a}	48.95 ±4.57^{a}	31.13 ±4.00^{b}	30.78 ±3.08^{b}	34.94 ±1.87^{b}
DSL(μ m/s)	16.49 ±3.52^{b}	16.29 ±1.16^{b}	28.37 ±4.23^{a}	30.12 ±1.23^{a}	24.56 ±4.33^{a}
WOB(%)	55.41 ±3.06^{b}	46.64 ±1.81^{c}	65.21 ±3.57^{a}	63.39 ±3.30^{a}	61.41 ±2.42^{a}
VAP(μ m/s)	54.05 ±10.30^{ab}	61.08 ±8.74^{a}	77.41 ±10.42^{a}	80.59 ±4.33^{a}	69.80 ±13.67a^{b}
LIN(%)	25.52 ±2.07^{c}	12.95 ±0.25^{d}	54.19 ±5.84^{a}	53.36 ±9.12^{a}	40.32 ±2.31^{b}
VSL(μ m/s)	25.93 ±5.51^{c}	25.52 ±2.34^{c}	57.91 ±9.37^{a}	61.13 ±7.02^{a}	40.28 ±7.61^{b}

Note: Different letters following means in the same row indicate significant differences using a one-way ANOVA followed by Duncan's multiple range test ($P < 0.05$).

Table S3. Effects of different concentrations of MYO on morphology and kinetic parameters of frozen-thawed sperm (Mean ± SEM, n=8)

	0(mM)	30(mM)	60(mM)	90(mM)	120(mM)
Curved tails(%)	5.80 ± 0.85^{a}	6.30 ± 1.22^{a}	3.47 ± 1.33^{a}	4.73 ± 1.07^{a}	4.00 ± 0.69^{a}
Coiled tails(%)	0.40 ± 0.26^{ab}	0.83 ± 0.51^{a}	0.43 ± 0.42^{c}	0.23 ± 0.25^{abc}	0.80 ± 0.72^{bc}
Distal plasmic drop(%)	5.57 ± 1.06^{a}	4.13 ± 0.21^{a}	4.77 ± 2.35^{a}	3.67 ± 1.50^{a}	4.53 ± 0.50^{a}
Proximal plasmic drop(%)	3.80 ± 2.51^{a}	2.83 ± 1.54^{a}	1.60 ± 0.66^{a}	2.53 ± 0.72^{a}	2.70 ± 1.39^{a}
VCL(μ m/s)	120.95 ± 12.59^{ab}	133.44 ± 4.10^{ab}	123.91 ± 16.29^{b}	136.93 ± 10.28^{ab}	130.47 ± 10.38^{a}
BCF（Hz）	31.36 ± 0.70^{ab}	33.53 ± 1.17^{ab}	38.63 ± 8.34^{a}	32.60 ± 0.30^{ab}	30.42 ± 0.87^{b}
DSL(μ m/s)	26.69 ± 3.56^{c}	27.37 ± 3.14^{bc}	21.34 ± 6.12^{c}	38.61 ± 0.79^{a}	34.53 ± 4.84^{ab}
WOB(%)	63.28 ± 4.15^{a}	59.73 ± 1.48^{a}	55.44 ± 10.88^{a}	65.47 ± 2.69^{a}	63.41 ± 1.86^{a}
VAP(μ m/s)	79.14 ± 8.90^{ab}	79.99 ± 3.30^{ab}	66.79 ± 13.54^{b}	96.14 ± 8.61^{a}	86.53 ± 9.47^{a}
LIN(%)	52.33 ± 3.98^{a}	44.89 ± 5.26^{a}	48.49 ± 3.25^{a}	49.40 ± 4.01^{a}	46.66 ± 5.67^{a}
VSL(μ m/s)	55.95 ± 9.18^{a}	56.07 ± 6.32^{a}	49.31 ± 11.34^{a}	62.89 ± 3.08^{a}	54.59 ± 7.64^{a}

Note: Different letters following means in the same row indicate significant differences using a one-way ANOVA followed by Duncan's multiple range test ($P < 0.05$).

Table S4. Effects of different concentrations of LP on morphology and kinetic parameters of frozen-thawed sperm (Mean ± SEM, n=8)

	0(mM)	50(mM)	100(mM)	150(mM)
Curved tails(%)	$6.43±1.29^{b}$	$16.40±8.58^{a}$	$1.70±0.66^{b}$	$7.47±3.07^{ab}$
Coiled tails(%)	$0.33±0.12^{b}$	$1.90±0.90^{a}$	$0.20±0.20^{b}$	$0.33±0.21^{b}$
Distal plasmic drop(%)	$11.10±3.52^{a}$	$8.40±3.35^{ab}$	$4.10±2.20^{b}$	$11.93±1.18^{a}$
Proximal plasmic drop(%)	$4.13±1.32a^{b}$	$9.20±5.86^{a}$	$2.27±1.04^{b}$	$4.43±1.69^{ab}$
VCL(μ m/s)	$133.55±9.53^{ab}$	$141.09±10.36^{a}$	$145.79±9.67^{a}$	$118.55±13.03^{b}$
BCF（Hz）	$36.62±5.03^{a}$	$34.95±2.85^{a}$	$32.51±1.56^{a}$	$31.46±0.82^{a}$
DSL(μ m/s)	$21.45±8.49^{a}$	$27.64±3.00^{a}$	$33.68±8.81^{a}$	$28.48±1.16^{a}$
WOB(%)	$53.87±7.54^{a}$	$60.16±4.61^{a}$	$63.74±8.63^{a}$	$60.19±3.63^{a}$
VAP(μ m/s)	$70.60±17.80^{b}$	$88.87±4.89^{ab}$	$97.65±14.28^{a}$	$76.78±5.77^{b}$
LIN(%)	$29.43±12.91^{a}$	$39.31±5.71^{a}$	$41.26±13.59^{a}$	$43.72±7.03^{a}$
VSL(μ m/s)	$43.37±6.84^{a}$	$51.83±3.75^{a}$	$57.97±13.30^{a}$	$53.74±3.75^{a}$

Note: Different letters following means in the same row indicate significant differences using a one-way ANOVA followed by Duncan's multiple range test ($P < 0.05$).

Table S5. Effects of three-drug combination addition on morphology and kinetic parameters of frozen-thawed sperm (Mean ± SEM, n=8)

	0(mol/L)	MYO(90 mM)	LP(100 mM)	Lim(150mM)	three-drug combination diluent
Curved tails(%)	5.78±1.63[a]	4.72±1.98[a]	5.50±1.78[a]	5.43±0.95[a]	5.20±2.42[a]
Coiled tails(%)	0.20±0.25[a]	0.32±0.64[a]	0.70±0.70[a]	0.55±0.82[a]	0.23±0.38[a]
Distal plasmic drop(%)	7.12±3.06[a]	6.80±3.09[a]	5.13±1.73[a]	7.45±3.54[a]	3.43±3.10[a]
Proximal plasmic drop(%)	5.85±1.04[a]	3.52±2.02[bc]	3.57±2.52[bc]	4.23±1.41[ab]	1.57±1.38[c]
VCL(μ m/s)	123.49±18.39[a]	125.75±7.80[a]	124.13±11.92[a]	124.88±17.92[a]	123.49±18.39[a]
BCF（Hz）	28.98±2.52[a]	33.50±3.39[a]	32.40±3.33[a]	32.12±3.96[a]	29.87±3.76[a]
DSL(μ m/s)	25.46±2.77[b]	27.47±5.53[b]	29.15±3.62[ab]	27.12±7.35[b]	36.82±8.87[a]
WOB(%)	56.25±2.85[b]	60.75±4.75[ab]	60.87±4.75[ab]	60.00±8.41[ab]	65.79±0.70[a]
VAP(μ m/s)	68.07±10.14[a]	77.68±12.83[a]	78.81±8.11[a]	71.63±19.24[a]	80.12±21.94[a]
LIN(%)	34.65±3.08[c]	42.23±3.74[bc]	48.80±7.72[ab]	45.28±15.80[ab]	53.57±3.71[a]
VSL(μ m/s)	51.55±21.57[a]	48.33±7.22[a]	54.26±5.17[a]	48.52±14.56[a]	64.14±14.77[a]

Note: Different letters following means in the same row indicate significant differences using a one-way ANOVA followed by Duncan's multiple range test ($P < 0.05$).

Table S6. Effects of different concentrations of Glycerin in combination addition on morphology and kinetic parameters of frozen-thawed sperm (Mean ± SEM, n=8)

	0%	1%	2%	3%
Curved tails(%)	24.20±2.46^{a}	19.30±7.72^{a}	6.93±3.01^{b}	5.20±2.42^{b}
Coiled tails(%)	2.57±0.74^{a}	1.47±0.61^{b}	0.62±0.71^{c}	0.23±0.38^{c}
Distal plasmic drop(%)	15.58±1.00^{a}	16.83±8.08^{a}	4.55±1.00^{b}	3.43±3.10^{b}
Proximal plasmic drop(%)	26.82±5.19^{a}	14.63±5.70^{b}	6.82±7.73^{c}	1.57±1.38^{c}
VCL(μ m/s)	86.59±18.83^{b}	66.32±17.98^{c}	128.92±16.76^{a}	127.30±19.32^{a}
BCF（Hz）	34.57±4.04^{a}	31.45±3.20a^{b}	29.35±2.40^{b}	29.87±3.76a^{b}
DSL(μ m/s)	13.45±4.15^{b}	12.17±4.24^{b}	36.11±10.38^{a}	36.82±8.87^{a}
WOB(%)	58.37±6.75^{a}	62.16±3.71^{a}	63.88±4.78^{a}	65.79±0.70^{a}
VAP(μ m/s)	34.04±14.06^{b}	22.99±5.97^{b}	85.12±17.90^{a}	80.12±21.94^{a}
LIN(%)	34.85±9.22^{b}	43.85±9.07^{ab}	52.89±8.99^{a}	53.57±3.71^{a}
VSL(μ m/s)	25.33±5.84^{b}	21.99±5.68^{b}	63.33±15.96^{a}	64.14±14.77^{a}

Note: Different letters following means in the same row indicate significant differences using a one-way ANOVA followed by Duncan's multiple range test ($P < 0.05$).
