# Supplementary material for: The Effects of Limonin, Myo-Inositol, and L-Proline on the Cryopreservation of Debao Boar Semen
Source: Animals (Basel). 2025 Jul 27;15(15):2204. doi: 10.3390/ani15152204 (PMC12345456; doi:10.3390/ani15152204)
Supplement: Supplementary file 1 [file animals-15-02204-s001.zip › Supplementary Materials-2.pptx]

## Slide 1
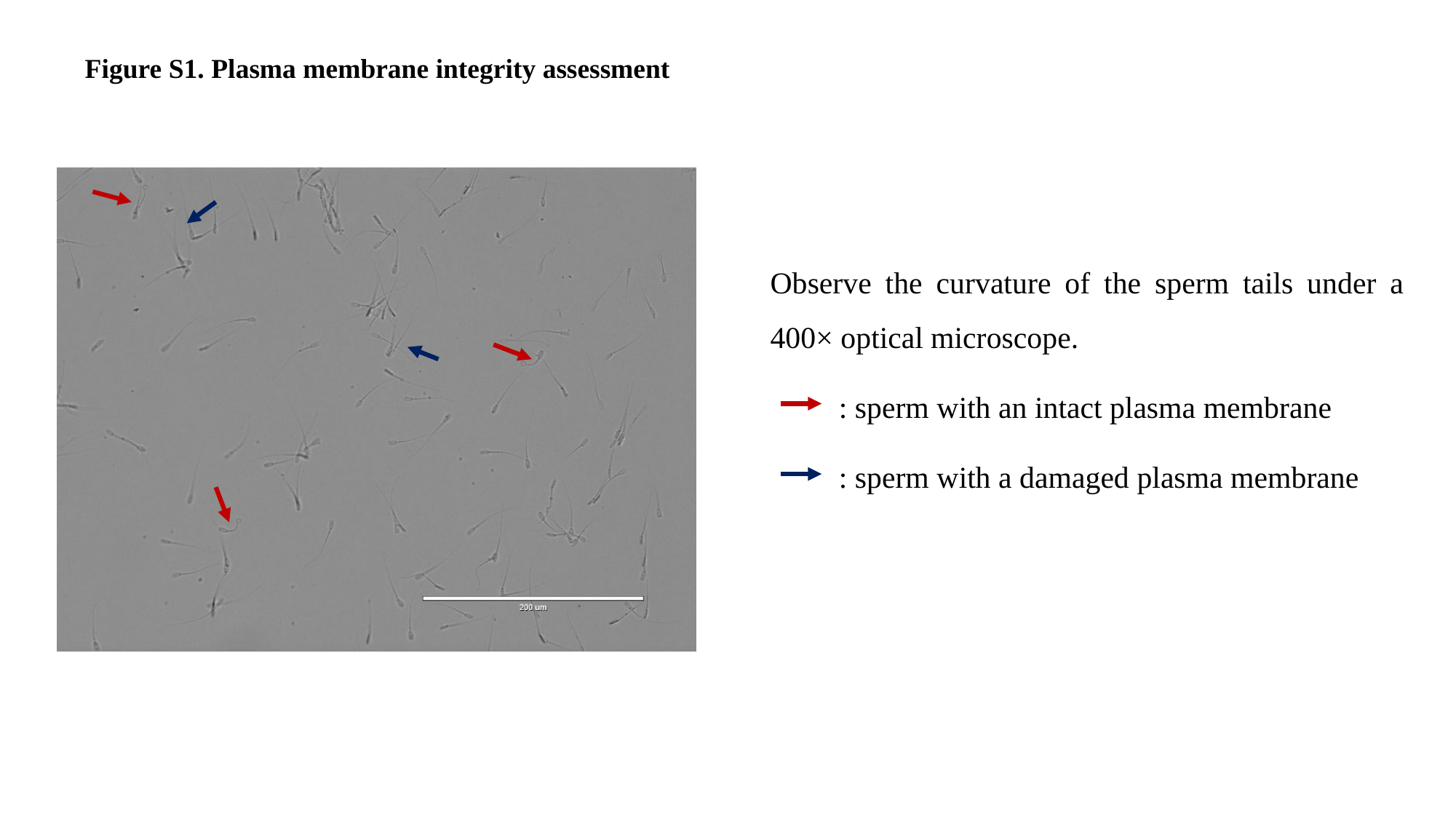

Figure S1. Plasma membrane integrity assessment
Observe the curvature of the sperm tails under a 400× optical microscope.
 : sperm with an intact plasma membrane
 : sperm with a damaged plasma membrane

## Slide 2
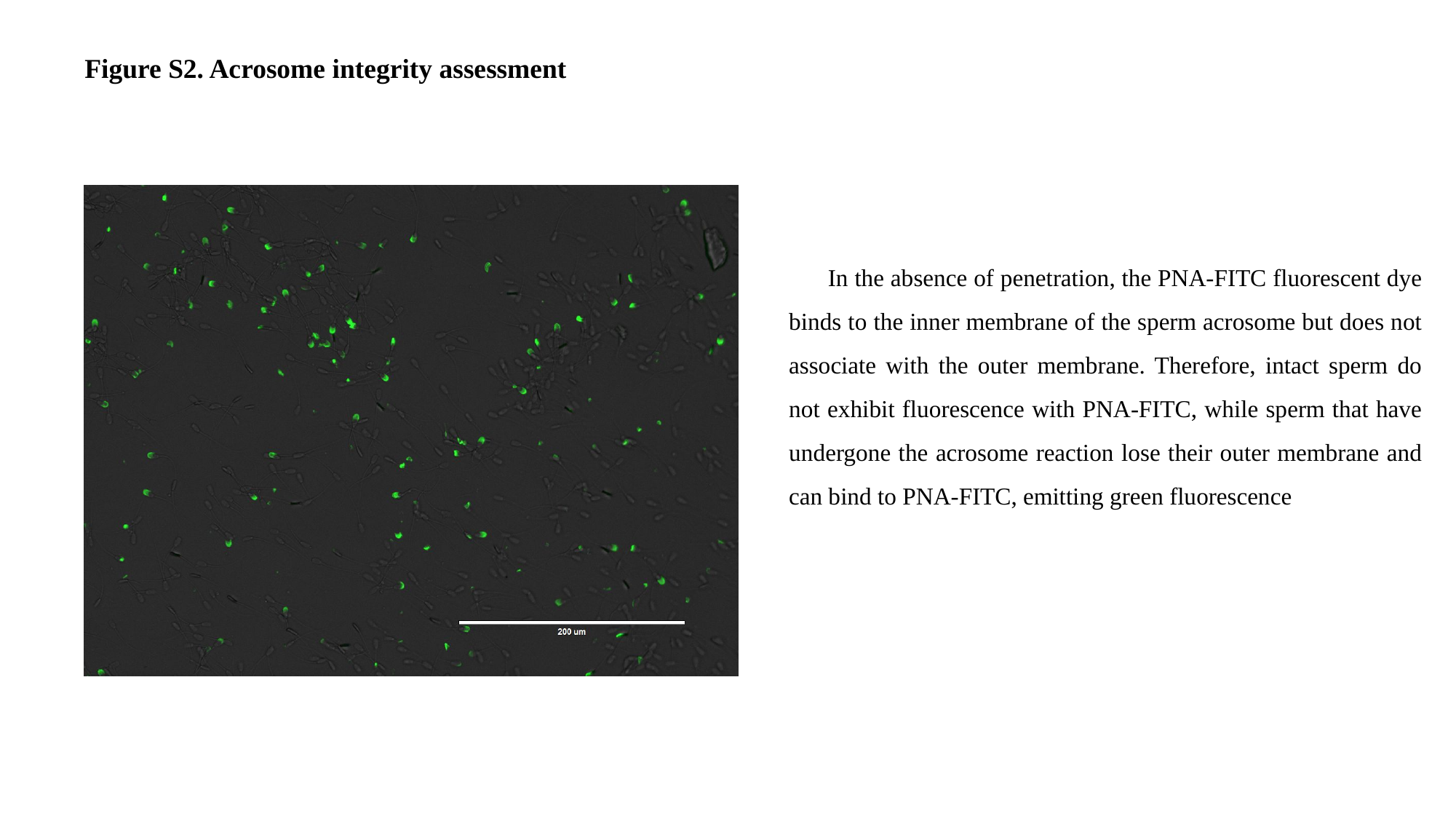

Figure S2. Acrosome integrity assessment
 In the absence of penetration, the PNA-FITC fluorescent dye binds to the inner membrane of the sperm acrosome but does not associate with the outer membrane. Therefore, intact sperm do not exhibit fluorescence with PNA-FITC, while sperm that have undergone the acrosome reaction lose their outer membrane and can bind to PNA-FITC, emitting green fluorescence

## Slide 3
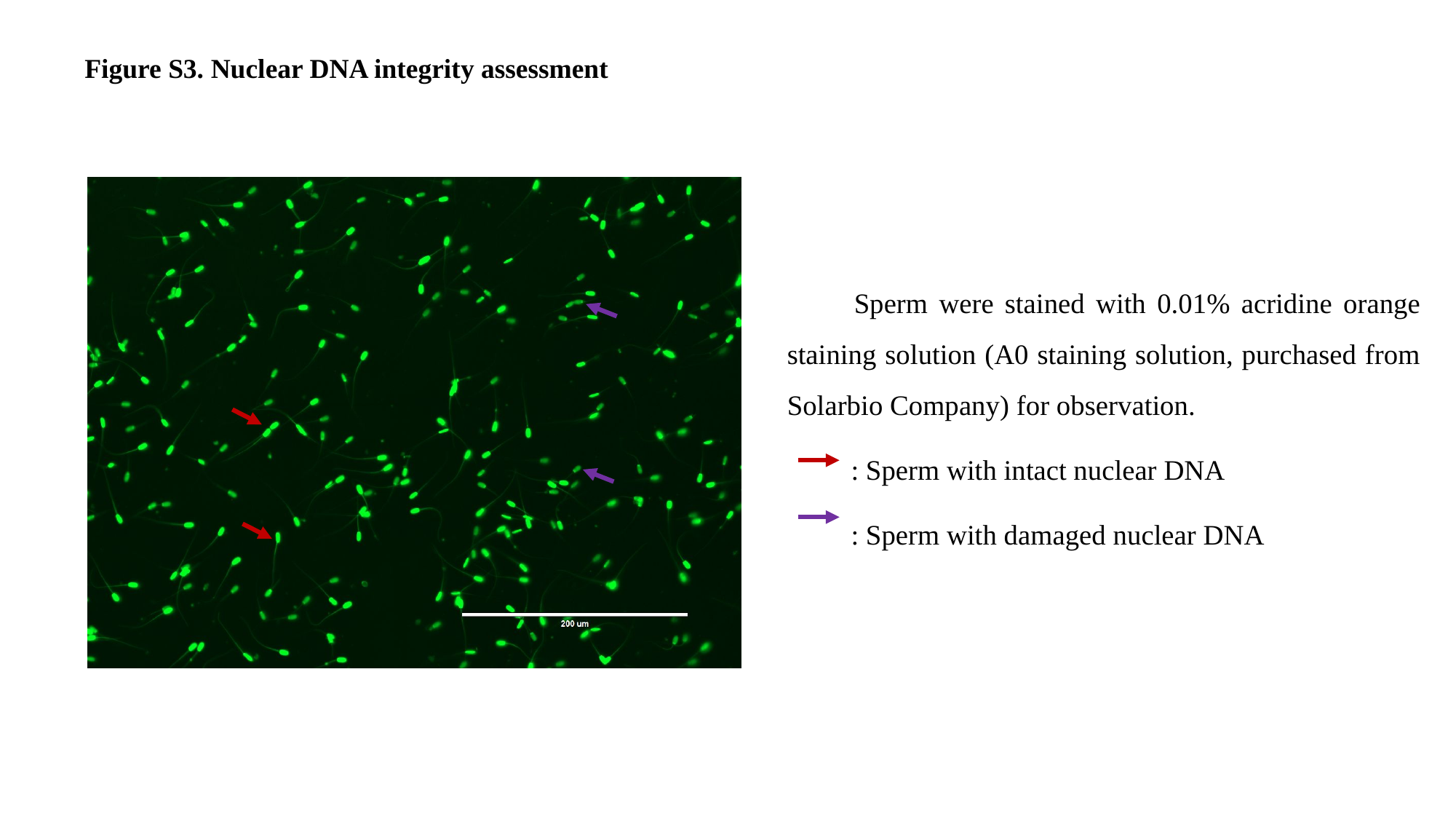

Figure S3. Nuclear DNA integrity assessment
 Sperm were stained with 0.01% acridine orange staining solution (A0 staining solution, purchased from Solarbio Company) for observation.
 : Sperm with intact nuclear DNA
 : Sperm with damaged nuclear DNA

## Slide 4
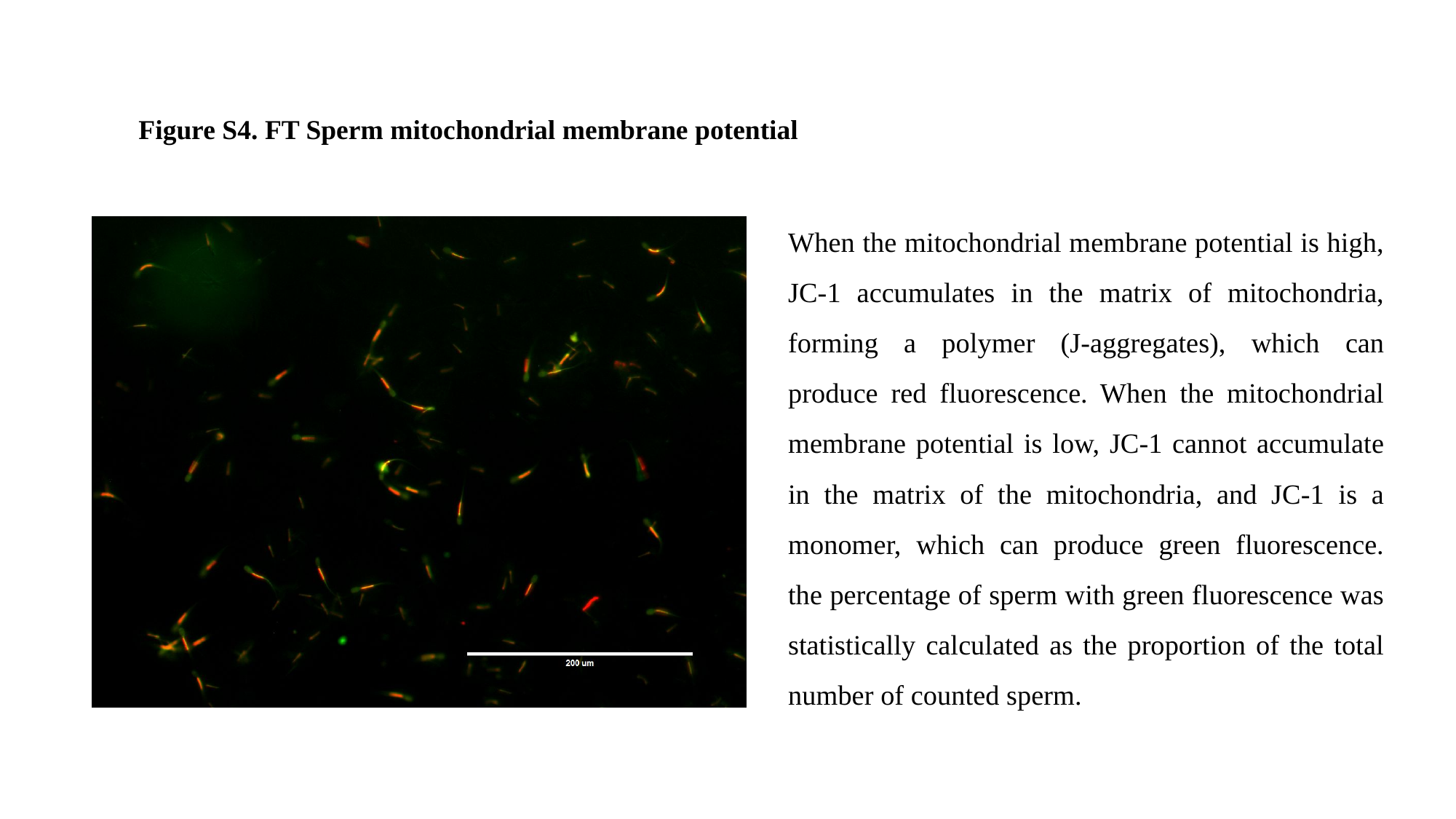

Figure S4. FT Sperm mitochondrial membrane potential
When the mitochondrial membrane potential is high, JC-1 accumulates in the matrix of mitochondria, forming a polymer (J-aggregates), which can produce red fluorescence. When the mitochondrial membrane potential is low, JC-1 cannot accumulate in the matrix of the mitochondria, and JC-1 is a monomer, which can produce green fluorescence. the percentage of sperm with green fluorescence was statistically calculated as the proportion of the total number of counted sperm.
